# Exploring perceived training and professional development needs of Australian dietetic students and practising dietitians in the area of eating disorders: a focus group study

**DOI:** 10.1186/s40337-022-00567-0

**Published:** 2022-03-18

**Authors:** Elizabeth Kumiko Parker, Mellisa Anne Ashley, Courtney Moretti, Deanne Maree Harris, Anita Stefoska-Needham

**Affiliations:** 1 Department of Dietetics and Nutrition, Westmead Hospital, P.O. Box 533, Wentworthville, Westmead, NSW 2145 Australia; 2 Sydney School of Health Sciences, Faculty of Medicine and Health, The University of Sydney, Sydney, NSW 2006 Australia; 3 Adult Eating Disorder Service, Western Sydney Local Health District, North Parramatta, NSW 2145 Australia; 4 SMART Foods Centre, Illawarra Health and Medical Research Institute, School of Medicine, Faculty of Science, Medicine and Health, University of Wollongong, Wollongong, NSW 2522 Australia; 5 Department of Nutrition & Dietetics, Tamworth Rural Referral Hospital, Tamworth, NSW 2340 Australia

## Abstract

**Background:**

Timely diagnosis and treatment of eating disorders (EDs) are essential for achieving the best possible outcomes, and dietitians have an important role in the multidisciplinary team. ED-specific training has been shown to enhance the knowledge, mental health literacy and confidence of health professionals in providing patient treatment. However, the ED-specific training needs of dietitians have yet to be determined. This study aimed to explore the perceived readiness of dietitians and student dietitians to treat patients with EDs; to identify the key training components that would enhance their confidence in delivering ED-specific treatment; and to examine any barriers associated with engagement in ED-specific professional development.

**Methods:**

A semi-structured question guide was developed by researchers to elicit information from six virtual focus groups consisting of a purposive sample of practising dietitians and student dietitians enrolled in their final year of an Australian tertiary accredited dietetic program. Members of professional organisations were approached to participate via email; and a recruitment flyer was promoted on various social media platforms. Discussions were recorded, transcribed verbatim and analysed qualitatively using inductive thematic analysis.

**Results:**

Thirty-eight participants (26 dietitians, 12 student dietitians) were recruited, mean age of 32.5 years ± 11 SD. Three major themes emerged: (1) reluctance to practice in EDs, which was associated with limited ED-specific training at university, lack of clinical guidelines, mental health complexities of patients with an ED, ambiguity regarding the dietitian’s role, systemic complexities with ED care, and beliefs of health professionals; (2) the need for additional ED-specific training and clinical supervision both during and after university, with the focus on identification, assessment, management and treatment, mental health literacy, and counselling skills, identified as an essential component to improving professional confidence and competence; (3) limited awareness and access/supply of ED-specific training opportunities were found, which included the financial cost of training/clinical supervision, and limited access to suitable clinical supervision.

**Conclusion:**

Dietitians currently practising in the workplace and dietetic students perceive that enhanced ED-specific training during university and after graduation is essential to work with patients confidently and competently with EDs. This research has implications for Australian university dietetic programs and workforce development.

**Plain English Summary:**

This study aimed to explore the perceptions and readiness of dietitians and student dietitians to treat patients with eating disorders (EDs); to identify the key training components that would enhance their confidence and competence in delivering treatment; and to examine any barriers associated with engaging in ED-specific professional development. Twenty-six dietitians and twelve dietetic students participated in six virtual focus group discussions, which identified three main themes: (1) reluctance to practice which was associated with limited ED-specific training at university, lack of clinical guidelines, mental health complexities of patients with an ED, ambiguity regarding the dietitian’s role, systemic complexities with ED care, and beliefs of health professionals; (2) the importance of engaging in ED-specific training, beyond an introductory level, during university and after graduation to confidently and competently work with patients with EDs; (3) barriers to accessing further ED-specific training and clinical supervision were found, including poor awareness of training opportunities, high financial cost of training/clinical supervision, and limited access to obtaining suitable clinical supervision. Results from this study provide insight into the ED-specific training needs of practising and student dietitians. This research has value for university programs and workforce development.

**Supplementary Information:**

The online version contains supplementary material available at 10.1186/s40337-022-00567-0.

## Introduction

Eating disorders (EDs), which include anorexia nervosa, bulimia nervosa, and binge eating disorder, are psychiatric illnesses, characterised by disturbances in eating behaviours, such as restrictive eating, binge-eating, and/or weight control behaviours including self-induced vomiting and laxative abuse [1, 2]. Other features include an intense fear of weight gain, preoccupation with eating, shape and weight, and body image disturbance [1, 2]. Biological, psychological and sociocultural factors all contribute to the aetiology of EDs [1], and if left untreated, EDs can result in a starvation-state with severe physical and psychological complications, including significantly lower quality of life and premature mortality [3–5].

In Australia, approximately 16% of the population is affected by an ED or disordered eating [6], with increasing prevalence of people engaging in disordered eating practices [7]. Additionally, many individuals are reluctant to disclose their ED behaviours to healthcare providers [8–10]. Previous research has indicated that some individuals may delay their treatment for up to 5 years after symptom onset [11]. As failure to provide early intervention has been associated with a longer illness duration, increased severity of illness and more severe physical health complications; timely diagnosis, early intervention and effective treatment, delivered by a skilled multidisciplinary team (MDT) can reduce the morbidity and mortality associated with an ED [12].

While clinical practice guidelines for the treatment of EDs recommend the involvement of dietitians in the multidisciplinary therapeutic team [13, 14], the specific role of the dietitian had not been described until recently [15]. The Australia & New Zealand Academy of Eating Disorders (ANZAED) have published dietetic-specific clinical practice and training standards [15]. According to these standards, dietitians are expected to take an active role in early identification and screening of at-risk individuals. Dietitians can also use their knowledge and skills to conduct a comprehensive nutrition assessment that identifies the severity of malnutrition, based on the physical and psychological signs and symptoms of starvation. Furthermore, dietetic assessment of an individual’s food beliefs, dieting practices, food avoidances and disordered eating behaviours, aid the nutrition diagnosis, and nutrition care plan [16]. The dietitian is also involved in the provision of psychoeducation, nutrition counselling, and monitoring progress towards treatment goals through ongoing evaluation. It has also been suggested that dietitians can assist with conducting behavioural experiments to gradually challenge food fears and/ or situations [17], dispel dietary myths, and support patients to rebuild a healthier relationship with food [15, 18].

While these new practice and training standards by ANZAED [15] acknowledge the important role of dietitians in the MDT, previous research indicates that dietitians and student dietitians report low confidence in treating patients with an ED [19, 20], and applying ED treatment modalities [20]. Inadequate university and postgraduate training, as well as limited clinical placement opportunities, have been identified as key factors affecting professional confidence [19, 21, 22], although a lack of mental health literacy may also play a role [19]. To improve confidence levels, participants in the study, published by Denman et al. [20], expressed a desire for further ED-specific training, however, the details of this training were not investigated. Therefore, the aim of this study was to explore the perceptions of dietetic students and practising dietitians towards their readiness to treat patients with EDs based on their training; to identify key training components that would enhance their confidence in delivering ED-specific treatment; and to examine any barriers associated with engagement in ED-specific professional development.

## Methods

### Study design

This qualitative descriptive study drew upon an interpretive phenomenological approach [23] to capture participants’ thoughts and feelings regarding the topic in everyday terms, using virtual focus group discussions. The reporting of findings adhered to the guidelines outlined in The Consolidated Criteria for Reporting Qualitative Research (COREQ) [24] (Additional file 1: Supplement 1). This study received ethics approval from University of Wollongong Human Ethics Committee (2020/063).

### Participants and recruitment

Participants were a purposive sample of student dietitians and practising dietitians residing in any state or territory within Australia. Inclusion criteria included dietetic students enrolled in their final year of an Australian accredited tertiary dietetic program, and practising dietitians eligible for membership of Dietitians Australia (DA). Dietitians and student dietitians who spoke English and had access to a computer with video-conferencing functionality, were able to participate. Individuals were approached through: (1) a professional email distribution through the membership networks of ANZAED and DA; and (2) the dissemination of a recruitment flyer through social media (The Mindful Dietitian and Dietitian Connection Facebook and LinkedIn pages, respectively). Participation was voluntary and participants could withdraw from the study at any time. All participants were asked to respond via email to express their interest in participating. They were sent a follow up email to confirm inclusion criteria, along with the participant’s information sheet and consent form. Consent forms were returned via email or post prior to the participant being recruited into a focus group.

### Data collection

A semi-structured question guide was developed by the authors for use in the focus groups assessing the following areas: perceived readiness to treat patients with EDs based on training; key training components that would enhance confidence delivering ED-specific treatment; and barriers to professional development (Additional file 2: Supplement 2). Participants were recruited to focus groups comprising of 6–10 individuals. Dietitians and student dietitians were recruited to separate focus groups. Focus group discussions were conducted virtually using the video-conferencing package Zoom [25], between April and June 2020. All focus groups were facilitated by two members of the research team (CM, EP). Focus group discussions lasted between 30 and 60-min each and were audio recorded and transcribed verbatim using Otter.au © (2021).

### Data analysis

All focus group transcripts were compared to the original audio recordings for accuracy. Transcripts were analysed inductively using Braun and Clarke’s six phases of thematic analysis [26]. Four researchers (CM, EP, MA, DH) independently read and familiarised themselves with the transcripts and manually coded the data. All researchers contributed to the discussion of codes and emerging themes until consensus agreement was reached on dominant themes. To establish the rigour of the study, the criteria proposed by Lincoln and Guba was used [27]. Confirmability was ensured through independent coding by multiple reviewers and a clear audit trail. Transferability was demonstrated by thick (detailed) descriptions of participants and the context [28], and credibility was demonstrated with the use of verbatim participant quotes.

## Results

Thirty-eight individuals, thirty-seven female and one male, with a mean age of 32.5 years (± 11 years), participated in one of the six focus groups held by the research team. Participants were recruited from New South Wales (50%), Queensland (26%), Victoria (10%), South Australia (10%) and Western Australia (4%). All participants were retained from recruitment to focus group.

Four focus groups comprised of practising dietitians (n = 26, 68%), with an average of 9.5 years’ (± 10.5 years) employment experience as a dietitian. Their employment settings included public hospitals (n = 13, 34%), private practice (n = 7, 18%), community health centres (n = 3, 7%), private hospital (n = 1, 3%), a non-government organisation (n = 1, 3%), with one new graduate seeking work (n = 1, 3%). Ninety two percent of practising dietitians (n = 24) had been exposed to patients with an ED within their employment history. The other two focus groups consisted of student dietitians in their final year of university study (n = 12, 32%) and of these, 42% (n = 5) had been exposed to patients with an ED during clinical placement.

Analysis of the data yielded three dominant themes which included: (1) reluctance to practice; (2) the need for additional ED-specific training and clinical supervision; (3) limited access/supply to ED-specific training and clinical supervision; including a number of associated subthemes (Table 1).

**Table 1:** Themes and subthemes

Themes	Subthemes
1. Reluctance to practice	(a) Limited ED-specific training at university (b) Lack of clinical guidelines (c) Mental health complexities of patients with an ED (d) Ambiguity regarding the role of the dietitian (e) Systemic complexities with ED care (f) Beliefs of health professionals
2. The need for additional ED-specific training and clinical supervision	(a) Need for diverse training activities (b) Professional shadowing is valuable (c) Clinical practice opportunities are important (d) Clinical supervision is imperative
3. Limited access/supply to ED-specific training and clinical supervision	(a) Lack of awareness of ED-specific training (b) Financial burden of training and clinical supervision (c) Limited access/supply of supervisors for clinical supervision

### Reluctance to practice

Student dietitians and practising dietitians reported a lack of training in the identification, assessment, management, and treatment of patients with EDs in their university programs. Inadequate training was linked to low professional confidence and a general reluctance to treat patients with EDs.

In early years … a barrier to me treating eating disorder patients was not feeling competent … and a part of that would include not recognising red flags well or … not assessing or asking the … most appropriate questions to identify eating disorders…Training at university was at best 1–2-hour lectures .... and readiness to treat eating disorder patients after graduating I'd say, very, very, very low… looking back now particularly I think at the time, I probably didn't know what I didn't know. (Participant 36, Dietitian).

Participants also perceived that there was insufficient content in accredited university programs and limited clinical guidance on working with the multifaceted needs of this patient group such as how to communicate about sensitive issues.

We … went over different kinds of eating disorders, but I feel like we didn't really get much guidance in how to approach patients with eating disorders … it didn't really feel like it was all that helpful for me anyway… I wouldn’t really know what to do (Participant 29, Student dietitian).

Further limitations in university curricula were described by participants, specifically a lack of practical learning activities, such as ED-specific case studies, which impeded their confidence to put skills into practice.

We weren't given a case study on it ... whereas we were for so many other different conditions. So, I think that maybe a case study would have been really helpful just to be able to delve into it a little bit more (Participant 31, Student dietitian).

Constraints with ED-specific clinical placement opportunities during their final year of university training also contributed to some participants feeling inadequately trained and lacking confidence to practice. Additionally, those participants who experienced exposure to patients with an ED during clinical placement stated that it was “only in an observational capacity”* (Participant 34, Dietitian).*

In terms of my personal clinical placement experience I didn’t have any eating disorder exposure whatsoever. (Participant 10, Dietitian).

Concerns were expressed by some participants regarding the distinct lack of published clinical guidelines to guide dietetic treatment of EDs, compared with other medical conditions, which contributed to their reluctance to practice. Additionally, the mental health complexities of working with this patient group were acknowledged.

We have entire semesters dedicated to a subject like diabetes and then they have such specific clinical guidelines as to how you can treat that patient, and eating disorders, because it's so closely tied in with mental health, is a bit more high stakes in my eyes, like there's not like a set of guidelines on how to treat that person. So, I feel like I would need more training in that area to be more confident. (Participant 18, Student dietitian).

Another factor that contributed to a reluctance to practice was confusion with respect to their scope of practice and the undefined role of the dietitian in the broader therapeutic team.

We find a lot of the pressure put on us to sort of run and be in charge of the admissions. So, it would be good to sort of have all your training on that, so you can be confident in saying where your scope lies. (Participant 20, Dietitian).

Systemic key challenges to providing ED care included the lack of a local clinical policy or defined treatment pathway. However, when steps were taken to mitigate these challenges, such as implementing a local clinical practice guideline, greater team cohesion was achieved, as articulated by one dietitian:

.. it has made the most massive difference, just to be able to get everyone on the team on the same page, from the time of admission and not talk through the same things until you're blue in the face every time (Participant 26, Dietitian).

Participants also stressed their concerns regarding the inequity of dietetic care provided to patients with an ED in the community. The lack of knowledge regarding government incentives for outpatient dietetic treatment of EDs by other health professionals were also reported as an issue in ED care contributing to dietitian reluctance to practice in EDs.

Coming from more the community private practice sort of space, we get a lot of pushbacks from GPs, especially writing the new [Medicare Eating Disorder Treatment] plans, because there's been so much unknown around that for the GPs… you get pushback from both other dietitians unwilling to see, and then GPs, and then psychologists within the private sector as well, and so when people do present asking for help, sometimes it will be the actual therapist pushing back and saying “no, let's just, let's talk about other stuff right now”, which I find as a dietitian, trying to work as a team is really disheartening as well. (Participant 6, Dietitian).

A lack of available funding in public health services, particularly in rural and regional areas of Australia, was reported. Specialised ED services, typically geographically located in large metropolitan areas, were often reported to have long patient waiting lists.

We haven’t got a specialised eating disorder unit, and so we often have bed blockages where we sort of manage and stabilise the acute decompensation and refeeding risk, and then we have a lot of challenges in getting the patients across to a specialised eating disorder unit (Participant 12, Dietitian).

Private sector treatment was frequently described as being too expensive for many patients with EDs, even with government funded incentives for outpatient care.

… there is private eating disorder support services available in our district, but the majority of our consumers just can't afford it. (Participant 7, Dietitian).

Participants described that other health professionals’ past negative experiences with treating hospitalised patients with an ED had led to a perceived avoidance of their responsibility in directing patients’ care.

… one of the main barriers is the patient's ambivalence about treatment seems to be reflected very much in the treating teams and also in the hospital and health services. They're often seen as very expensive to treat and very difficult to treat. I see hospitals that will not admit them. Consultants who don't want to treat them, health services who want to push them through the system as quickly as possible, community dietitians who feel that it's not within their brief to treat their eating disorders patients and have actually been directed not to treat eating disorders diagnoses. There is a lot of discriminatory behaviour that we've seen out there. (Participant 3, Dietitian).

Furthermore, the MDT’s ambivalence towards the treatment of ED-specific symptoms in the presence of other comorbidities was identified as a key challenge for dietetic practice in both inpatient and outpatient settings.

When they've got a history of eating disorders, but their main reason for admission is something completely different … the medical team just wants to treat whatever the acute problem is and the eating disorder sort of gets overlooked. (Participant 25, Dietitian).

### The need for additional ED-specific training and clinical supervision

The second major theme that emerged was the need for additional ED-specific training, beyond an introductory level, during both university training and after graduation, to improve confidence and competence. Key training needs identified by participants included identification, assessment, management, and treatment of patients with an ED, mental health literacy, and counselling skills.

Participants identified a variety of ED-specific online and face-to-face training activities that have contributed to building their confidence for working with patients with an ED.

I did some face to face [training] with ANZAED last year in October. And then another one day [training] with the DA just earlier this year…. For me, doing those extra trainings definitely built my confidence a lot more. Even if I'm not fully ready to do more complex cases, that's fine by me, you know, but even just being able to screen and assess and then going “okay, well this is not at my level yet, I will give you to somebody more specialised”, so I found it super helpful. (Participant 11, Dietitian).

The practice of shadowing was reported as valuable for hospital dietitians who had the opportunity to work alongside a senior dietitian and observe patient care.

What I found most helpful since graduating is training from dietitians. So, lots of work shadowing, observing patient care and the way MDT is run, and then lots of debriefing about different situations that may potentially occur and ways to manage or respond to those. (Participant 21, Dietitian).

Clinical opportunities to engage with patients with EDs, to practice and develop newly acquired skills, was reported as equally as important as observational opportunities.

It's a more complex process, and they are reluctant to get new grads involved but I think it's just more experience and that more exposure would be pretty helpful in preparing new grads. (Participant 34, Dietitian).

Clinical supervision was viewed as essential for improving confidence for working with patients with an ED, although participants appeared to struggle to describe specifically the process of clinical supervision. For some participants, clinical supervision was more important at the start of their career working with patients with EDs than it was later.

I would say it is important to me 10 out of 10 and I don’t believe that I should be working in the area of eating disorders without having received the supervision and mentoring that I did prior to undertaking that workload. (Participant 21, Dietitian).

Hospital dietitians in particular reported more value in peer review over a more formalised arrangement like clinical supervision due to the timeliness of feedback.

In the clinical setting…informal clinical supervision is much more valuable… If you have a question, you're not sure what way to go about it, you're going to need to kind of know what to do quite soon. Whereas if we've got that formal clinical supervision, maybe you meet with your senior once a month or once every couple of months. (Participant 10, Dietitian).

Senior dietitians raised the value of interdisciplinary supervision.

Overall as a team… I think there's definitely value in having that interdisciplinary clinical supervision as well … I certainly found that gave me much more leaps and bounds and suddenly kept me in check for my level of stress and burnout potential (Participant 8, Dietitian).

Clinical supervision was valued by student dietitians, although they did not always distinguish it from mentorship or direct line management. According to some student dietitians, clinical supervision, rather than a general recommendation, should be considered mandatory when working with patients with an ED.

I think [clinical] supervision is invaluable and absolutely required, but I don't really think it's really spoken about enough at uni either [and] when you first head out it should be non-negotiable (Participant 27, Student dietitian).

### Limited access/supply to ED-specific training and clinical supervision

The third major theme that emerged was limited access to suitable ED-specific training and clinical supervision. Some study participants had engaged in a wide range of training opportunities to upskill in preparation for working with patients with an ED. However, others were not aware of specific ED-training opportunities and resources they could access.

There are a lot of resources out there, but … dietitians who have just graduated aren’t even aware of the resources that are there for them (Participant 3, Dietitian).

Maybe a suggested pathway of what types of further training or supervision opportunities might be available for anyone who's interested in upskilling more in eating disorder situations, would be pretty handy to say, you know, these are the courses available and it would be a good idea to start with counselling or binge eating disorder or whatever (Participant 34, Dietitian).

The high cost of available ED-specific training was of particular concern to student participants and dietitians who were not employed in a government health service where training expenses were more likely to be fully or partially subsidised.

Having free access is important for you know students and for brand new dietitians as well. Because a lot of the time, there’s not much cash to start splurging on a whole lot of training. (Participant 27, Student dietitian).

The high cost of continuing professional development activities, including clinical supervision, was considered prohibitive for many clinicians, especially those practising in more than one clinical area, or in general outpatient settings where they would rarely encounter a patient with an ED.

Often there's quite significant costs attached to those online courses, they may be hundreds of dollars and you know, for example, and [it can] cost $70 per session to sit in group supervision. (Participant 3, Dietitian).

I think it's [professional development activities] even more expensive if it's not your entire caseload…it's a lot to put out $500 for a type of patient that you might see every, you know, couple of months. (Participant 4, Dietitian).

Difficulties with locating an appropriately experienced clinical supervisor, was a barrier to engaging in clinical supervision, especially in rural or regional settings of Australia.

There’s very few dietitians who are around who have actually got enough experience in working in eating disorders and have had training in supervision as well to provide supervision. (Participant 3, Dietitian).

Many student dietitians were unsure how to locate a clinical supervisor or mentor with experience in EDs and would have to rely primarily on connections made during clinical placements or connections made in future workplaces. Despite attending dietitian networking events such as workshops and conferences, many student dietitians expressed feelings of awkwardness and apprehension when approaching experienced dietitians.

To be honest, I just feel really nervous about approaching someone [to be a supervisor], that’s a big part of it. I don’t want to be a hassle. I find it really daunting thinking about it. (Participant 27, Student dietitian).

## Discussion

In this Australian study, currently practising dietitians and final year dietetic students perceived that ED-specific training, beyond an introductory level, is essential for them to confidently and competently work with patients with EDs, and to reduce their reluctance to practice in this clinical area. A reluctance to treat patients with EDs by dietitians is problematic as there is an unmet demand for treatment in this patient group [9]. Furthermore, with improved treatment pathways and recent increases to government incentives for outpatient ED treatment [29, 30], dietitians may be more likely to encounter patients with an ED within any clinical setting. Similarly, dietitians may be more likely to see patients with an undiagnosed ED, with many patients likely to opt to seek treatment for symptom management, even in the absence of a formalised ED diagnosis [9, 31, 32].

In the present study, participants reported some confusion about their role, for example, hospital-based dietitians frequently described coordinating patients’ care, and despite acknowledging that this is not within their scope of practice, this was not always communicated well with other health professionals. Therefore, in addition to dietitians clearly understanding their own scope of practice and role [33], it is essential that open and collaborative communication pathways are established between all members of the therapeutic team and roles within the team are clearly defined [34, 35].

In the outpatient setting, the role of the dietitian and scope of practice have also been ambiguous, with the majority of ED treatment manuals failing to endorse the inclusion of a dietitian in the multi-disciplinary treatment approach [36–38]. Furthermore, prior to the recent development of the consensus-based guidelines for outpatient dietetic treatment of EDs by McMaster et al. [39], existing guidelines lacked specific clinical practice guidance for dietitians [13, 14, 40]. Inconsistencies among ED specialists regarding the need for a dietetic referral for assessment, education, and nutritional guidance in patients, have also added to the confusion around scope of practice [39]. The recently published ANZAED practice and training standards for dietitians [15], may alleviate some of the ambiguity surrounding the role of the dietitian in treating patients with EDs as both inpatients and outpatients. However, it is currently unknown how widely disseminated and utilised these ANZAED standards are, or what their impact has been on the perceived confidence and competence levels of dietitians, and their willingness to treat patients with EDs. The recent development of the ANZAED ED credentialing system, may address these issues, with the recognition of minimum qualifications in knowledge, training, and professional development activities to provide a minimum acceptable standard of care [41].

Participants in this study identified several gaps in ED-specific training, notably related to university curricula. Both practising dietitians and student dietitians in this study perceived that addressing these deficits would better prepare them for clinical work with patients with an ED. Study findings indicate that more lecture content focussing on identifying, assessing, managing and treating EDs and disordered eating would be beneficial. Enhancement of the mental health literacy of dietetic students, and acknowledgment of the scope of practice and the role of the dietitian within the treating team, were also highly valued by the study participants. Similarly, the incorporation of comprehensive practical learning opportunities via case studies, role-playing or simulations. Additional opportunities for reflective practice, advanced clinical placements, and opportunities to interact within a MDT were also recommended by participants. Increased communication and collaboration between academics and clinicians engaging in ED research, patient/carer advocacy groups, individuals with a lived experience of an ED, and university dietetic course convenors and lecturers, may also address these existing gaps. This in turn may facilitate increased awareness of and uptake of the ANZAED practice and training standards [15] into university curricula. Similarly, these collaborations may also enhance awareness of available post-graduate ED-specific training for anyone who is interested in working clinically after graduation.

Counselling and communications skills were identified as key areas requiring further development through post-graduation training, consistent with research by Whisenant et al. [42] who found that 79.3% of surveyed dietitians who work with patients with EDs, report a desire for further training in counselling theory and techniques. Online learning modules represent a cost-effective method of enhancing knowledge, skills and confidence for mental health providers including dietitians [43], and this mode of training delivery has been shown to appeal to student and practising dietitians [20]. Previous research has also shown that mental health literacy is enhanced through ED-specific training, which may reduce the stigmatisation and discrimination by any health professionals towards patients with EDs [43, 44].

Clinical supervision has been shown to improve knowledge and skills, and prevent stress and burnout for clinicians when regularly encountering patients with EDs [22, 45, 46]. After graduation, participants in this study acknowledged the value of clinical supervision for working with patients with EDs, although the majority of hospital-based dietitians preferred an informal or ad-hoc approach to clinical supervision, compared to a more formalised supervision arrangement. However, Kirk et al. [45] have recommended a more formalised clinical supervision arrangement to enable clarification between mentoring, direct line management, and clinical supervision, which was not always clearly differentiated among student dietitians in the current study. Additionally, senior dietitians expressed interdisciplinary supervision as an invaluable tool in expanding skills for treating patients with EDs. However, this was rarely pursued by participants due to financial constraints, despite being acknowledged as a significant resource for professional support and learning [47]. Recommendations to include interdisciplinary supervision as detailed in this study, may enhance the diverse range of skills of dietitians, aid the delineation of roles within the MDT, and acknowledge boundaries between disciplines [22]. Online clinical supervision sessions, provided in a small-group format, may be one opportunity for less expensive clinical supervision opportunities, without any geographical constraints.

Several limitations were encountered during this study, particularly the risk of bias, inherent in purposive sampling through the selected distribution methods, and self-selection bias of participants with greater interest or exposure to patients with EDs. Data saturation may not have been reached due to the sample size. Hence, it is possible that practising dietitians and student dietitians not included in this study may offer differing perspectives to those described. Nevertheless, the strength of this study was the diversity of participants with differing clinical experience levels, employment settings and geographical locations within Australia. Future research exploring the perspectives of course conveners and lecturers of accredited university dietetic programs is recommended to identify further gaps in tertiary education and post-graduate training for dietitians who wish to work clinically after graduation.

## Conclusion

In this study, dietitians currently practising in the workplace and final year dietetic students report a reluctance to treat patients with EDs based on ambiguity regarding their role in the broader therapeutic team, and see the need for additional ED-specific training both during and after university to confidently and competently work with patients with EDs. Reducing access barriers to effective training and clinical supervision would assist with enhancing confidence and competence for working in EDs. The broader dissemination of professional development resources such as dietetic position papers and ANZAED practice and training standards may assist in the enhancement of knowledge and skill development. This research has implications for Australian university dietetic programs and workforce development.

## Supplementary Information

**Additional file 1. Supplement 1**. Consolidated criteria for reporting qualitative studies (COREQ): 32-item checklist.

**Additional file 2. Supplement 2**. Semi-structured question guide used in focus groups.

## Data Availability

The datasets used and/or analysed during the current study are available from the corresponding author on reasonable request.

## Abbreviations

ANZAED: The Australia and New Zealand Academy of Eating Disorders
DA: Dietitians Australia
ED: Eating disorder
MDT: Multidisciplinary team

## References

[1] Treasure J, Duarte TA, Schmidt U (2021). Eating disorders. Lancet.

[2] American Psychiatric Association (2013). Diagnostic and statistical manual of mental disorders.

[3] Jahraus J (2018). Medical complications of eating disorders. Psychiatr Ann.

[4] Agh T, Kovacs G, Supina D, Pawaskar M, Herman BK, Voko Z, Sheehan DV (2016). A systematic review of the health-related quality of life and economic burdens of anorexia nervosa, bulimia nervosa, and binge eating disorder. Eat Weight Disord.

[5] Arcelus J (2011). Mortality rates in patients with anorexia nervosa and other eating disorders. Arch Gen Psychiatry.

[6] Hay P, Girosi F, Mond J (2015). Prevalence and sociodemographic correlates of DSM-5 eating disorders in the Australian population. J Eat Disord.

[7] Hay PJ, Mond J, Buttner P, Darby A (2008). Eating disorder behaviors are increasing: findings from two sequential community surveys in South Australia. PLoS ONE.

[8] Hudson JI, Hiripi E, Pope HG, Kessler RC (2007). The prevalence and correlates of eating disorders in the National Comorbidity Survey Replication. Biol Psychiatry.

[9] Hart LM, Granillo MT, Jorm AF, Paxton SJ (2011). Unmet need for treatment in the eating disorders: a systematic review of eating disorder specific treatment seeking among community cases. Clin Psychol Rev.

[10] Forrest LN, Smith AR, Swanson SA (2017). Characteristics of seeking treatment among U.S. adolescents with eating disorders. Int J Eat Disord.

[11] Hamilton A, Mitchison D, Basten C, Byrne C, Byrne S, Goldstein M, Hay P, Heruc G, Thornton C, Touyz S. Understanding treatment delay: perceived barriers preventing treatment-seeking for eating disorders. Aust N Z J Psychiatry. 2021. 10.1177/00048674211020102 34250844

[12] Lang K, Glennon D, Mountford V, McClelland J, Koskina A, Borwn A, et al. Early intervention for eating disorders. In: Wade T (ed) Encyclopedia of feeding and eating disorders. Singapore: Springer Singapore; 2016. p. 1–6.

[13] Hay P, Chinn D, Forbes D, Madden S, Newton R, Sugenor L, Touyz S, Ward W (2014). Royal Australian and New Zealand College of Psychiatrists clinical practice guidelines for the treatment of eating disorders. Aust N Z J Psychiatry.

[14] National Institute for Health and Care Excellence. NICE Guidelines, eating disorders: recognition and treatment [Internet]. NICE; 2017. [cited 2021 February 17]. https://www.nice.org.uk/guidance/ng69.

[15] Heruc G, Hart S, Stiles G, Fleming K, Casey A, Sutherland F, Jeffrey S, Roberton M, Hurst K (2020). ANZAED practice and training standards for dietitians providing eating disorder treatment. J Eat Disord.

[16] Academy of Nutrition and Dietetics. Abridged nutrition care process terminology (NCPT) reference manual. Chicago, IL: Academy of Nutrition and Dietetics; 2017.

[17] Ashley M, Crino N (2010). A novel approach to treating eating disorders in a day-hospital treatment program. Nutr Diet.

[18] Mittnacht AM, Bulik CM (2015). Best nutrition counselling practices for the treatment of anorexia nervosa: a Delphi study. Int J Eat Disord.

[19] Ozier A, Henry B (2010). Preliminary report on dietitians’ views and confidence related to nutrition care for eating disorders. ICAN Infant Child Adolesc Nutr.

[20] Denman E, Parker EK, Ashley MA, Harris DM, Halaki M, Flood V, Stefoska-Needham A (2021). Understanding training needs in eating disorders of graduating and new graduate dietitians in Australia: an online survey. J Eat Disord.

[21] Trammell E, Reed D, Boylan M (2016). Education and practice gaps of registered dietitian nutritionists working with clients with eating disorders. Top Clin Nutr.

[22] Cairns J, Milne RL (2006). Eating disorder nutrition counseling: strategies and education needs of English-speaking dietitians in Canada. J Am Diet Assoc.

[23] Sandelowski M (2010). What’s in a name? Qualitative description revisited. Res Nurs Health.

[24] Tong A, Sainsbury P, Craig J (2007). Consolidated criteria for reporting qualitative research (COREQ): a 32-item checklist for interviews and focus groups. Int J Qual Health Care.

[25] Zoom Video Communications Inc. Security guide; 2021*.* Zoom Video Communications Inc. https://explore.zoom.us/docs/doc/Zoom-Security-White-Paper.pdf. Accessed 21 Nov 2021.

[26] Braun V, Clarke V (2006). Using thematic analysis in psychology. Qual Res Psychol.

[27] Lincoln YS, Guba EG (1986). But is it rigorous? Trustworthiness and authenticity in naturalistic evaluation. New Dir Program Eval.

[28] Ponterotto JG (2006). Brief note on the origins, evolution, and meaning of the qualitative research concept “thick description”. Qual Rep.

[29] NSW Ministry of Health. NSW service plan for people with eating disorders (2021–2025); 2021. SHPN (MH) 210144 ISBN 978-1-76081-637-7.

[30] Australian Government Department of Health. Medicare Benefits Schedule: Eating Disorders—Quick Reference Guide; 29/10/2019 [cited 2021 Nov 22]. http://www.mbsonline.gov.au/internet/mbsonline/publishing.nsf/Content/773AA9AA09E7CA00CA2584840080F113/$File/Eating%20Disorders%20Quick%20Reference%20Guide%2029Oct2019.pdf.

[31] Gulliksen KS, Nordbo RHS, Espeset EMS, Skarderud F, Holte A (2015). The process of help-seeking in anorexia nervosa: patients’ perspective of first contact with health services. Eat Disord.

[32] Ogg EC, Millar HR, Pusztai EE, Thom AS (1997). General practice consultation patterns preceding diagnosis of eating disorders. Int J Eat Disord.

[33] Hart S, Russell J, Abraham S (2011). Nutrition and dietetic practice in eating disorder management. J Hum Nutr Diet.

[34] Saloff-Coste CJ, Hamburg P, Herzog DB (1993). Nutrition and psychotherapy: collaborative treatment of patients with eating disorders. Bull Menninger Clin.

[35] McDevitt S, Passi V (2018). Evaluation of a pilot interprofessional education programme for eating disorder training in mental health services. Ir J Psychol Med.

[36] Lock J, Le Grange D (2015). Treatment manual for anorexia nervosa.

[37] Fairburn CG (2008). Cognitive behavior therapy and eating disorders.

[38] McMaster CM, Wade T, Franklin J, Hart S (2020). A review of treatment manuals for adults with an eating disorder: nutrition content and consistency with current dietetic evidence. Eat Weight Disord.

[39] McMaster CM, Wade T, Franklin J, Hart S (2020). Development of consensus-based guidelines for outpatient dietetic treatment of eating disorders: a Delphi study. Int J Eat Disord.

[40] Yager J, Devlin M, Halmi K, Herzog D, Mitchell J, Powers P (2014). Guideline watch (August 2012): practice guideline for the treatment of patients with eating disorders. Focus.

[41] McLean SA, Hurst K, Smith H, Shelton B, Freeman J, Goldstein M, Jeffrey S, Heruc G (2020). Credentialing for eating disorder clinicians: a pathway for implementation of clinical practice standards. J Eat Disord.

[42] Whisenant SL, Smith BA (1995). Eating disorders: current nutrition therapy and perceived needs in dietetics education and research. J Am Diet Assoc.

[43] Maguire S, Li A, Cunich M, Maloney D (2019). Evaluating the effectiveness of an evidence-based online training program for health professionals in eating disorders. J Eat Disord.

[44] Brownlow RS, Maguire S, O’Dell A, Dias-da-Costa C, Touyz S, Russell J (2015). Evaluation of an online training program in eating disorders for health professionals in Australia. J Eat Disord.

[45] Kirk SFL, Eaton J, Auty L (2000). Dietitians and supervision: should we be doing more?. J Hum Nutr Diet.

[46] Howard V, Eddy-Imishue GK (2020). Factors influencing adequate and effective clinical supervision for inpatient mental health nurses' personal and professional development: an integrative review. J Psychiatr Ment Health Nurs.

[47] The British Dietetic Association. BDA Practice Supervision. The British Dietetic Association: Birmingham; 2011 [cited 2021 February 17]. https://www.bda.uk.com/uploads/assets/42fd0e92-7d14-4d59-856c8b83eb339007/practice-supervision-document.pdf.

