# Supplementary material for: Exploring perceived training and professional development needs of Australian dietetic students and practising dietitians in the area of eating disorders: a focus group study
Source: J Eat Disord. 2022 Mar 18;10:40. doi: 10.1186/s40337-022-00567-0 (PMC8934001; doi:10.1186/s40337-022-00567-0)
Supplement: Supplementary file 2 — Additional file 2. Supplement 2. Semi-structured question guide used in focus groups. [file 40337_2022_567_MOESM2_ESM.docx]

**Supplement 2. Semi-structured question guide used in focus groups.**

| **Focus group for dietitians:**   1. How many years’ experience do you have working as a dietitian? 2. What experience have you had treating people with eating disorders? 3. Describe any barriers to you treating patients with eating disorders. 4. Describe your experience with eating disorder training at university. 5. Describe your perceived readiness to treat patients with eating disorders after graduating from university. 6. What additional training in eating disorders, if any, have you had since graduating? 7. Describe your experience with post graduate eating disorder training. 8. Describe your perceived readiness to treat patients with eating disorders after completing further postgraduate eating disorder training. 9. What do you see as key components of training in eating disorders when you finish university? 10. What has been your experience with professional/clinical supervision? 11. How important is professional/clinical supervision to you? 12. Are there any barriers experienced with professional/clinical supervision? 13. Is there anything else you would like to add that may help assist your professional development in eating disorders?   **Focus group for student dietitians**:   1. Describe your experience with eating disorder training at university. 2. Describe your perceived readiness to treat patients with eating disorders after graduating from university. 3. What would help increase your confidence and skills to work with patients who have an eating disorder? 4. What do you see as key components of training in eating disorders when you finish university? 5. How would you prefer the training to be delivered? 6. Are there any areas/topics that you would find most helpful when learning about eating disorders? 7. How important is professional/clinical supervision to you once graduating? 8. Are there any barriers to seeking professional/clinical supervision once graduating? 9. Is there anything else you would like to add that may help assist your professional development in eating disorders? |
| --- |
